# A rare presentation of esthesioneuroblastoma: a case report and review of the literature

**DOI:** 10.11604/pamj.2023.46.111.42205

**Published:** 2023-12-21

**Authors:** Ahmed Ben Sghier, Soumiya Samba, Meriem Bouabid, Soufiane Berhili, Mohammed Moukhlissi, Loubna Mezouar

**Affiliations:** 1 Radiotherapy Department University Mohamed VI Hospital, Faculty of Medicine and Pharmacy, Mohamed First University Oujda, Oujda, Morocco

**Keywords:** Esthesioneuroblastoma, surgical resection, adjuvant radiotherapy, case report

## Abstract

Esthesioneuroblastoma is a rare malignant tumor developing from the olfactory neuroepithelium. It represents less than 5% of all cancers of the nasal cavity. We are going to report the observation of a patient followed at the regional oncology center of Oujda in Morocco who presented a locally advanced esthesioneuroblastoma. Treatment consisted of surgical resection followed by adjuvant radiotherapy on the tumor bed. Currently, the patient is in good control of his disease.

## Introduction

First described by Berger and Richard in 1924 [1], esthesioneuroblastoma has the characteristic of being a rare malignant tumor of the nasal cavities, arising from neuroepithelial elements of the olfactory mucosa. Esthesioneuroblastoma is also known as esthesioneurocytoma or neuroepithelioma, or more commonly, olfactory neuroblastoma. The diagnosis is based on histological and immunohistochemical analysis, with surgery and radiotherapy being the main therapeutic options. Olfactory neuroblastoma is characterized by a slow progression with a high recurrence rate even after a long period of remission. We report a clinical case of an esthesioneuroblastoma treated at the regional oncology center in Oujda with a review of the literature on this tumor location.

## Patient and observation

**Patient information:** a 52-year-old patient, professional tailor, tobacco sniffer for 20 years, with no personal or family history of cancer. He consulted following a nasal obstruction with episodes of mild epistaxis lasting for 8 months. These symptoms occurred in the context of headaches and weight loss estimated at 12 kg over 3 months.

**Clinical findings:** the clinical examination found a conscious patient, hemodynamically and respiratory stable. Examination of the nostrils revealed a protruding mass obstructing the right nasal cavity. The remainder of the physical examination was normal.

**Figure 1:**
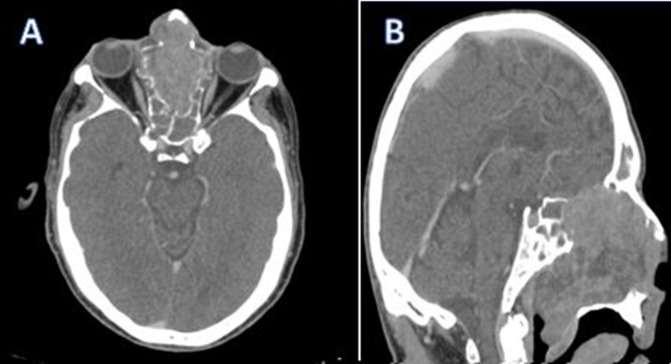
computed tomography scan in axial (A) and sagittal (B) sections with injection of the contrast product showing an esthesioneuroblastoma with total destruction of the cribriform plate of the ethmoid

**Figure 2:**
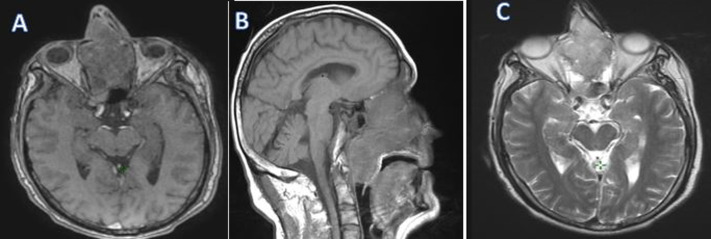
T1 in axial (A), sagittal (B) and T2 in axial (C) magnetic resonance imaging sections showing an esthesioneuroblastoma occupying all of the nasal cavities and extending to the oropharynx

**Figure 3:**
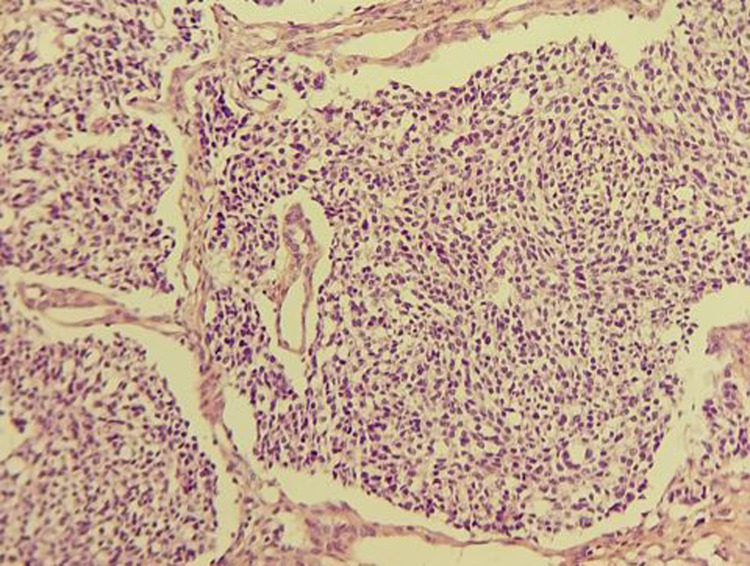
histological appearance of tumor proliferation arranged in lobules and clusters of monomorphic cells compatible with esthesioneuroblastoma

**Diagnostic assessment:** a naso-fibroscopy was carried out, revealing a total obstruction of the right nasal cavity by a tumor process and a left septal deviation preventing passage. The computed tomography (CT) and magnetic resonance imaging (MRI) confirm that it is a large tumor process measuring 73*54*40mm, centered on the right nasal cavity with irregular contours in places, which enhances heterogeneously after intravenous injection of contrast product, without secondary localization (Figure 1, Figure 2). The biopsy of the mass with the anatomopathological and immunohistochemical study made it possible to make the diagnosis of esthesioneuroblastoma grade III according to the Hyams classification (Figure 3).

**Therapeutic intervention:** the patient benefited from partial surgical resection of the tumor and adjuvant radiotherapy to the nasal cavity and cervical lymph node areas at a total dose of 50 Gy in 25 fractions with a boost on the tumor bed of 20 Gy in 10 fractions using intensity modulated radiotherapy technique.

**Figure 4:**
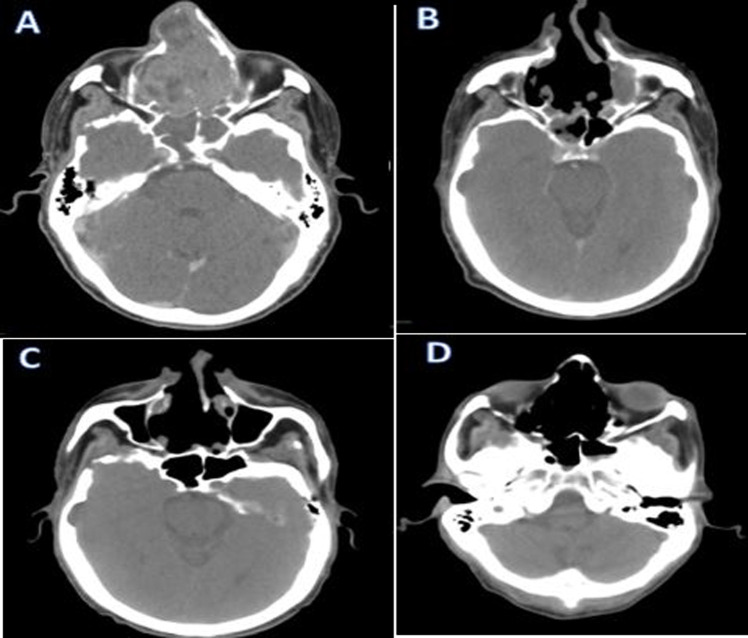
computed tomography scan axial section before tumor resection (A), after tumor resection (B), after radiotherapy (C) and after 6 months from the end of treatment (D)

**Follow-up and outcomes:** the operative suites and adjuvant radiotherapy took place without any major incident. After 6 months of progress, the patient is still alive and in good control of his disease (Figure 4).

**Patient perspective:** the patient was delighted with the quality of care.

**Informed consent:** it was obtained from the patient for the publication of this case report.

## Discussion

Esthesioneuroblastoma (ENB) is a rare entity, accounting for 3% of all endonasal cancers [2]. The true frequency of this type of cancer is difficult to assess due to advancements in histological techniques that improve the ability to diagnose these lesions. However, the estimated incidence of esthesioneuroblastoma is 0.4 cases per million inhabitants [3]. It can occur at any age with a peak incidence in the fifth decade, showing a slight male predominance. There is no clear correlation between ENB and risk factors, family history of cancer, or genetic predisposition. The exact origin of this tumor and the type of cells it involves are still debated. Proposed anatomical sites of origin for this tumor include Jacobson's organ or vomeronasal organ, the sphenopalatine ganglion, parasympathetic nerve endings of the nasal mucosa, and most importantly, the olfactory placode [4]. The indicative symptoms of ENB are non-specific and depend on the local and distant extension of the tumor. Symptoms are often unilateral and dominated by rhinological signs such as nasal obstruction, epistaxis, rhinorrhea, and sometimes anosmia. Less common signs include headaches, visual disturbances, exophthalmos, tearing, and behavioral changes indicating advanced disease. Secretory ENBs have been described, as causing paraneoplastic syndromes like Cushing's syndrome, malignant hypercalcemia, or hyponatremia due to inappropriate secretion of antidiuretic hormone [5]. The tumor appears as a polypoid intranasal mass on endoscopic examination, dark red or gray, friable, and bleeding upon contact. Clinical examination should be complemented by ophthalmological and neurological examinations due to the proximity of the tumor to optical structures and the base of the skull. Examination of cervical lymph nodes is important due to the lymphophilic nature of ENB. However, the non-specific symptoms and insidious progression of the tumor often lead to a late diagnosis.

Standard sinus radiography does not provide specific elements for diagnosis. It may show sinus opacity, calcifications, or changes in neighboring bones. Currently, CT and MRI are the necessary radiological examinations for assessing endonasal tumors. On CT bone window images, the tumor appears as a polypoid, hypodense mass with calcifications and bone destruction depending on the stage, particularly affecting adjacent structures like the cribriform plate, ethmoid roof, and medial orbital wall. Enhanced parenchymal CT images show a heterogeneously enhancing mass, with hypodense areas corresponding to necrotic zones. The hourglass appearance on coronal sections is typical of locally advanced tumors, featuring three parts: two wide sections (superior endocranial and inferior endonasal) and a narrow central part at the cribriform plate. On MRI, the tumor appears as hypo- or iso signal on T1-weighted images (more pronounced in necrotic areas) and iso- to hypersignal on T2-weighted images, with clear enhancement on contrast-enhanced sequences [6]. Magnetic resonance imaging is essential for assessing tumor resectability, and clarifying tumor extension to adjacent structures such as the anterior cranial fossa, retro-maxillary space, and orbital cavity. It also aids in optimizing radiation planning by facilitating target volume delineation and identifying organs at risk. The involvement of regional lymph nodes is assessed clinically, with combined CT and MRI, and ideally positron emission tomography (PET)-CT scans. For distant extension assessment, ideally, thoraco-abdominal CT scans should be conducted to detect hepatic and/or pulmonary metastases. Based on this assessment and the degree of tumor extension to adjacent structures, the tumor can be classified into three stages according to Kadish *et al*. initial classification in 1976, distinguishing localized nasal cavity tumors, nasosinusal tumors, and tumors extending beyond the nasosinusal cavities [5]. Kadish's classification was later modified and replaced by Foote *et al*. classification, adding a fourth stage for tumors with cervical or distant lymph node extension [7].

The diagnosis of esthesioneuroblastoma relies on histopathological and immunohistochemical studies. Several biopsies need to be performed to enable a thorough examination of the tumor and its various morphological features. Microscopically, esthesioneuroblastoma typically presents with nests of cells within a richly vascularized fibrillary stroma, along with more or less circumscribed lobules, in varying proportions. The tumor cells appear small, round, monomorphic, with rounded nuclei and scant cytoplasm. This tumor proliferation is characterized by the presence of rosettes or neuroblast-like pseudo-rosettes: Homer Wright rosettes in 30% of cases and Flessner-Wurtenstein pseudo-rosettes in 70% of cases. Based on the number of mitoses, nuclear polymorphism, types of rosettes, and the presence of mitosis, Hyams described four differentiation stages ranging from well-differentiated indolent forms to aggressive undifferentiated forms [8]. Immunohistochemical analysis shows diffuse positivity for neuroendocrine differentiation markers such as Neuron-Specific Enolase (NSE), synaptophysin, and chromogranin. Variable expression is observed for vimentin, cytokeratins, CD56, and PS-100, aiding in guiding the diagnosis [9]. The proliferation marker Ki 67 reveals a variable index ranging from 10% to 50% depending on the grade. Esthesioneuroblastomas are extremely rare tumors, which is why there is no standardized treatment approach for these tumors. The combination of surgery and radiotherapy is the most commonly used approach due to its better outcomes. Historically, the first surgical approach was trans-facial surgery, aiming to hollow out the lateral mass of the ethmoid bone while preserving the olfactory nerve fibers traversing the cribriform plate of the ethmoid bone (the anatomical origin of the tumor). In the 1980s, the trans-craniofacial approach emerged, with the primary goal of en-bloc tumor resection and achieving surgery with clear margins, this was well confirmed by the increase in overall survival and improvement of local control. However, the morbidity of the trans-craniofacial approach cannot be neglected.

With the development of endoscopic nasal surgery, endoscopic tumor resection has become relevant. The approaches vary among teams: some perform endoscopic resection of nasal tumors with anterior craniotomy, while others opt for endoscopic resection of nasal tumors and the roof of the ethmoid bone without craniotomy. Results from trials comparing the effectiveness of endoscopic surgery and craniofacial surgery are inconsistent due to limited patient numbers, short follow-up duration, and selection biases. Generally, authors recommend endoscopic resection for small tumors and craniofacial surgery for stage C and D tumors, allowing the surgeon to choose the technique they are most skilled in to ensure complete resection. Another major therapeutic modality is radiotherapy, often used as adjuvant therapy, sometimes neoadjuvant, or exclusively. Among the first trials evaluating the value of radiotherapy after surgery is the study by Foote *et al*. which did not show any superiority of the combined treatment compared to surgery alone [7]. However, the author points out that the patients treated with the combined treatment initially presented with more advanced tumors and higher stages. According to Dulguerov's meta-analysis, the 5-year survival rate following surgery alone was 48%, while it was 37% for radiotherapy alone. In contrast, survival after combined treatment (surgery and radiotherapy) was 68% [4]. These results are confirmed by several subsequent works such as the work of Ward in 2009 whose disease-free survival at 15 years was 83% in patients treated with surgery and radiotherapy, against 23% at 5 years and 0% at 15 years in patients treated with surgery alone [9]. This same study suggests that postoperative radiotherapy on the tumor bed contributes significantly to reducing the risk of distant lymph node metastases. According to certain literature data, the only circumstance justifying surgery without complementary radiotherapy would be for Kadish A-stage tumors with complete surgical resection. Generally, the dose varies between 50 Gy and 70 Gy in classical fractionation: between 50-60 Gy after complete resection and healthy limits or between 60-70 Gy after partial resection. Sometimes radiotherapy is used preoperatively to reduce the size of the tumor and facilitate tumor resection. However, the efficacy of neoadjuvant radiotherapy remains to be confirmed by more solid literature data. Exclusive radio-chemotherapy finds its place in elderly patients, in poor general condition, or refusing surgery, it is associated with very limited survival rates.

In cases involving lymph node involvement, authors recommend lymph node dissection combined with cervical irradiation. However, there's no consensus regarding preventive irradiation of cervical lymph node areas. For some authors, prophylactic irradiation of cervical lymph node areas leads to a reduction in cervical lymph node relapse with no impact on survival. This preventive cervical irradiation can be discussed for stage C and D tumors which present a significant risk of lymph node metastasis. Three-dimensional conformal radiotherapy was the most used technique for treating ENBs, but the tumor size and proximity to neural and optic structures made optimal target volume irradiation challenging. Intensity-modulated radiotherapy is a viable alternative, limiting dose to critical organs without compromising tumor control, associated with better survival and less radiation toxicity [10]. Proton therapy is also considered safe and effective for ENB treatment, preserving neighboring healthy tissues. Since the 1990s, the role of chemotherapy in the therapeutic arsenal of ENBs has been a topic of debate. Initially used neoadjuvantly before radiotherapy and then adjuvantly for stages C and D. However, the survival rates obtained were comparable with or without chemotherapy and the impact of chemotherapy on remission rates remains unclear. Chemotherapy is not always recommended in the treatment of ENBs, it is most appropriate in advanced stages in combination with other modalities, in high-grade lesions, in recurrent or non-operable tumours, and metastatic forms. Targeted therapy has shown promising results in isolated cases of recurrent or progressing ENB, especially with tyrosine kinase inhibitors, epidermal growth factor receptor (EGFR) inhibitors, and mTOR inhibitors. Despite therapeutic progress, the prognosis for ENBs remains poor, with estimated survival rates of around 50% at 5 years and 30% at 10 years, along with an increased risk of local and metastatic recurrence. Clinical stage at diagnosis, lymph node involvement, and histological grade according to the Hyams classification appear to be the main prognostic factors for ENBs. Clinical and radiological long-term follow-up of patients is necessary due to the significant risk of recurrence, even in later stages.

## Conclusion

Esthesioneuroblastoma (ENB) is a rare naso-sinus tumor. Complete surgical resection followed by adjuvant radiotherapy constitutes the therapeutic standard for localized tumors. Endoscopic resection is increasingly gaining ground, but further studies and longer patient follow-up will be necessary. The challenge of radiation toxicity can be overcome with the emergence of new radiotherapy techniques. The role of chemotherapy, especially adjuvant chemotherapy and targeted therapy, is yet to be determined. ENB is associated with significant recurrence rates, even in later stages, underscoring the need for long-term clinical and radiological patient follow-up.
